# Scientific Items

**Published:** 1885-07-20

**Authors:** 


### SCIENTIFIC ITEMS.

**An Educated Chimpanzee.**—I was once the owner of a highly educated chimpanzee. He knew all the friends of the house, all our acquaintances, and distinguished them readily from strangers. Every one treating him kindly he looked upon as a personal friend. He never felt more comfortable than when he was admitted to the family circle and allowed to move freely around, and open and shut doors, while his joy was boundless when he was assigned a place at the common table, and the guests admired his natural wit and practical jokes. He expressed his satisfaction and thanks to them by drumming furiously on the table. In his numerous moments of leisure his favorite occupation consisted in investigating carefully every object in his reach: he lowered the door of the stove for the purpose of watching the fire, opened drawers, rummaged boxes and trunks and played with their contents, provided the latter did not look suspicious to him. How easily suspicion was aroused in his mind might be illustrated by the fact that, as long as he lived, he shrank with terror from every common rubber-ball. Obedience to my orders and attachment to my person, and to everybody caring for him, were among his cardinal virtues, and he bored me with his persistent wishes to accompany me. He knew perfectly his time for retiring, and was happy when some one of us carried him to the bedroom like a baby. As soon as the light was put out he would jump into the bed and cover himself, because he was afraid of the darkness. His favorite meal was supper with tea, which he was very fond of, provided it was largely sweetened and mixed with rum. He sipped it from the cup, and ate the dipped bread-slices with a spoon, having been taught not to use the fingers in eating; he poured his wine from the bottle and drank it from the glass. A man could hardly behave himself more gentlemanlike at table than did that monkey.—From the "Ways of Monkeys," by Dr. Alfred E. Brehm, in *Popular Science Monthly* for June.

**Industrial Use of Natural Gas.**—The practical application of natural gas, as an article of fuel, to the purpose of manufacturing glass, iron, and steel, promises to work a revolution in the industrial interests of America—promises to work a revolution; for, notwithstanding the fact that, in many of the largest iron, steel, and glass factories in Pitsburg and its vicinity, natural gas has already been substituted for coal, the managers of some such works are shy of the new fuel, mainly for two reasons:

1. They doubt the continuity and regularity of its supply.

2. They do not deem the difference between the price of natural gas and coal sufficient, as yet, to justify the expenditure involved in the furnace changes necessary to the substitution of the one for the other. These two objections will doubtless disappear with additional experience in the production and regulation of the gas supply, and with enlarged competition among the companies engaging in its transmission from the wells to the works. At present the use of natural gas as a substitute for coal in the manufacture of glass, iron, and steel, is in its infancy.—From "The Fuel of the Future," by George Wardman, in *Popular Science Monthly* for June.

**Rates of Increase of Negro Population.**—Such being the history of the negroes in *ante bellum* days, when they were property, and when every consideration of self-interest prompted their owners to watch over their health, to encourage child-bearing, and to protect and preserve the children, is it to be supposed for a moment that this careless, improvident, ignorant race, thrown suddenly upon its own resources, should at once, or within a generation, take on a rate of increase more rapid than before emancipation? The wonder is, that in the past twenty years they have not fallen further behind.

Considering the colored race in this country as a whole, it is seen that it has not held its own, either in a state of slavery, or thus far in freedom. It is but another illustration of the fact, that an inferior race cannot thrive side by side with a superior one. It seems, therefore, under the circumstances, more profitable to study ways and means for preserving and strengthening the manual labor element of the South, rather than to debate the methods of getting rid of it.—From "Are we to become Africanized?" by Henry Gannet, in *Popular Science Monthly* for June.

**The Medical Electric Lamp.**—The electric lamp used for examining General Grants' throat is manufactured by agents of the Edison Light Company. It is mounted on a hard rubber holder, about seven inches long, having a reflector at the lamp end, by which the light can be thrown to any desired angle. The holder is connected by two silk-covered wires to a small storage battery carried in the pocket of the physician. The light is turned on by simply pressing a small button on the rubber holder, and the quantity is governed by another button convenient to the operator. The lamp is inserted in the mouth almost to the palate, with the reflector above the lamp, which throws the light down the throat. The lamp has no unpleasant heat, and gives a light equal to halt a sperm candle. The extreme simplicity of the whole appliance makes it very valuable to the physician and dentist.—*Scientific American.*

**To remove** old hard varnish, French polish, or paint from furniture or other wooden surfaces make a mixture consisting of five parts of a 36 per cent. solution of silicate of sodium or potassium, 1 part of a 40 per cent. solution of sodic hydrate, and 1 part caustic ammonia, and apply to the surface with a brush. Simple rinsing with water finishes the operation. This furnishes the subject of a recent German patent, and an experiment with it has demonstrated to the writer its efficiency with old varnish at least.—*National Druggist.*

